# Effect of ultrasound-guided continuous erector spinae plane block on postoperative pain and inflammatory response in patients undergoing modified radical mastectomy for breast cancer: study protocol for a randomised controlled trial

**DOI:** 10.1186/s13063-023-07777-0

**Published:** 2024-01-15

**Authors:** Liang Yu, Xiao-Juan Shen, He Liu, Yu-Ting Zhou, Qin Zhang, Zhen-Duo Zhang, Shu-Min Shen

**Affiliations:** 1 Department of Anesthesiology, Huzhou Central Hospital, The Fifth School of Clinical Medicine of Zhejiang Chinese Medical University, The Affiliated Huzhou Hospital, Zhejiang University School of Medicine, Affiliated Central Hospital, Huzhou University, No. 1558 Sanhuan North Road, Huzhou, 313000 Zhejiang China; 2 Department of 706A Ward Nursing, Huzhou Central Hospital, The Fifth School of Clinical Medicine of Zhejiang Chinese Medical University, The Affiliated Huzhou Hospital, Zhejiang University School of Medicine, Affiliated Central Hospital, Huzhou University, No. 1558 Sanhuan North Road, Huzhou, 313000 Zhejiang China; 3 Department of Breast Surgery, Huzhou Central Hospital, The Fifth School of Clinical Medicine of Zhejiang Chinese Medical University, The Affiliated Huzhou Hospital, Zhejiang University School of Medicine, Affiliated Central Hospital, Huzhou University, No. 1558 Sanhuan North Road, Huzhou, 313000 Zhejiang China

**Keywords:** Erector spinae plane block, Nerve block, Perioperative analgesia, Modified radical mastectomy, Cytokines, Post-mastectomy pain syndrome, Chronic pain after breast cancer surgery

## Abstract

**Background:**

A single injection of local anaesthetic (LA) in the erector spinae plane block (ESPB) can reduce pain after modified radical mastectomy (MRM) surgery, but the duration of analgesia is affected by the duration of the LA. The aim of this study is to investigate the effect of continuous ESPB on acute and chronic pain and inflammatory response after MRM surgery.

**Methods:**

In this prospective, randomised, controlled trial, we will recruit 160 patients, aged 18–80 years, scheduled for elective MRM surgery under general anaesthesia. They will be randomly assigned to two groups: a continuous ESPB group (group E) and a sham block group (group C). Both groups of patients will have a nerve block (group C pretended to puncture) and an indwelling catheter fixed prior to surgery. Electronic pumps containing LA are shielded. The primary outcome is the total consumption of analgesic agents. The secondary outcomes include the levels of inflammation-related cytokines; the occurrence of chronic pain (post-mastectomy pain syndrome, PMPS); static and dynamic pain scores at 2, 6, 12, 24 and 48 h postoperatively; and post-operative and post-puncture adverse reactions.

**Discussion:**

Analgesia after MRM surgery is important and chronic pain can develop when acute pain is prolonged, but the analgesic effect of a nerve block with a single injection of LA is limited by the duration of drug action. The aim of this trial is to investigate whether continuous ESPB can reduce acute pain after MRM surgery and reduce the incidence of chronic pain (PMPS), with fewer postoperative analgesic drug-related complications and less inflammatory response. Continuous ESPB and up to 12 months of follow-up are two innovations of this trial.

**Trial registration:**

Chinese Clinical Trial Registry (https://www.chictr.org.cn/) ChiCTR2200061935. Registered on 11 July 2022. This trial is a prospective registry with the following registry names: Effect of ultrasound-guided continuous erector spinae plane block on postoperative pain and inflammatory response in patients undergoing modified radical mastectomy for breast cancer.

**Supplementary Information:**

The online version contains supplementary material available at 10.1186/s13063-023-07777-0.

## Introduction

### Background and rationale {6a}

Breast cancer is the leading cause of cancer-related deaths among women worldwide, with a standardised incidence rate of 47.8 per 100,000 and a standardised mortality rate of 13.6 per 100,000 in 2020 [1]. The current treatment of choice for breast cancer is surgery, and modified radical mastectomy (MRM) surgery remains the most common surgical treatment modality [2]. MRM surgery involves the breast and axillary region, with large surgical incisions, and is prone to acute and chronic post-operative pain, which is one of the main factors causing stress and inflammatory reactions. Now, “post mastectomy pain syndrome” (PMPS) has become the main term to represent chronic pain persisting for at least 3 months after breast cancer-related surgery; the incidence of PMPS can range from 25 to 60% [3]. Patients with PMPS consume significantly more intraoperative or postoperative intravenous and oral analgesic medication and have more severe acute postoperative pain [3, 4]. Therefore, perioperative complex regional nerve blocks, implementation of multimodal analgesia, reduction of intravenous and oral analgesic drug consumption and enhanced acute pain management have positive significance in preventing PMPS, reducing the incidence of perioperative complications, alleviating stress and inflammatory responses and improving prognosis.

Regional analgesia techniques have been widely accepted by anaesthetists as the basis for multimodal analgesia. The erector spinae plane block (ESPB) is a novel regional block technique that was first reported in 2016 by Forero et al. [5] to be successfully applied to the treatment of thoracic neuropathic pain with good efficacy. Studies have shown that injecting 20 ml of 0.5% ropivacaine into the deep surface of the erector spinae fascia at the level of the T5 transverse process blocks the spinal nerve running there and blocks the ipsilateral T3-T9 spinal innervation area [5, 6]. A number of studies have shown good postoperative analgesia with ESPB in MRM surgery [7–9]. However, most current studies were conducted with a single injection of local anaesthetic (LA), and the duration of analgesia was limited by the duration of the LA. The perforator interface of the ESPB is free of important blood vessels and organs, and ultrasound-guided indwelling catheters for continuous ESPB are feasible. Therefore, we designed this trial to investigate the effect of ultrasound-guided continuous ESPB on postoperative pain and inflammatory response in patients undergoing MRM surgery for breast cancer. It is anticipated that continuous injection of LA to prolong the duration of analgesia will reduce the degree of acute post-operative pain and the incidence of chronic pain and decrease the degree of inflammatory response by continuously blocking the transmission of injurious stimuli.

## Objectives {7}

We hypothesised that ultrasound-guided continuous ESPB would reduce the amount of analgesic medication used during and after surgery and reduce the level of pain during catheter retention, thereby reducing the short-term side effects associated with anaesthesia and decreasing the incidence of post-surgical inflammatory reactions and chronic pain. To test this hypothesis, two groups of patients undergoing MRM surgery will be compared: group E received a continuous ESPB and group C received a sham puncture. The main aim of this study is to test the hypothesis that continuous nerve block overcomes the temporal limitations of a single injection of drug and provides longer analgesia, thereby reducing analgesic drug consumption, inflammatory response and the incidence of PMPS.

## Trial design {8}

This study will be conducted as a prospective, single-centre, double-blind, parallel-group, randomised, controlled trial.

## Methods: participants, interventions and outcomes

### Study setting {9}

The study will be conducted in patients undergoing elective MRM surgery for breast cancer in Huzhou Central Hospital, Zhejiang, China.

### Eligibility criteria {10}

The inclusion and exclusion criteria for participants in this study are as follows:

Inclusion criteria:

1. Scheduled to undergo the elective MRM surgery for breast cancer under general anaesthesia
2. American Society of Anesthesiologists (ASA) score of Ito III
3. Female aged 18–80 years with capacity
4. Agree to participate in this study and sign informed consent

Exclusion criteria:

1. Long-term use of opioids or other analgesics
2. Known hypersensitivity to the study medication (ropivacaine)
3. Severe mental illness and difficulty communicating
4. Liver or renal insufficiency
5. Without informed consent
6. History of breast surgery

### Who will take informed consent? {26a}

The eligibility of participants will be determined jointly by the anaesthesia and breast surgeons of the study team at our hospital. Written informed consent will be obtained from each study participant 1 day prior to surgery to allow sufficient time for participants to consider and voluntarily choose to participate in this study.

### Additional consent provisions for collection and use of participant data and biological specimens {26b}

Prior to obtaining informed consent from the participant, we will explain the method of puncture for the continuous ESPB and describe the pre-, intra- and post-operative data and venous blood that need to be asked for and collected. The details of what needs to be asked and collected will also be listed in the informed consent form. We will collect 3 ml of venous blood at a predetermined time point for plasma inflammatory cytokine and ropivacaine concentration assay. The blood sample will be sent to the central laboratory of our hospital. After centrifugation, the serum will be stored in test tubes at − 80 °C. Plasma levels of the inflammatory markers tumour necrosis factor (TNF)-α, interleukin (IL)-6 and IL-10 will be quantified using commercial enzyme-linked-immunosorbent serologic assay (ELISA) kits. Use high-performance liquid chromatography to determine the plasma concentration of ropivacaine.

### Interventions

#### Explanation for the choice of comparators {6b}

We describe all interventions as receiving continuous ESPB combined with general anaesthesia in full during the signing of the informed consent form. A sham nerve block is used for the comparator. In group C, only the erector spinae muscle is scanned with an ultrasound probe, followed by fixation of the line for the injection of the drug in the back and placement of the same electronic drug injection pump as in group E. In all patients, the electronic drug injection pump is wrapped in a black bag and kept for 48 h. The risks of ropivacaine infusion and systemic toxicity will be communicated to patients during the informed consent interview.

#### Intervention description {11a}

##### Preparing

All participants are routinely fasted for 8 h prior to the procedure and wait in the surgical preparation room for the nerve block operation while receiving oxygen and monitoring a 5-lead ECG, non-invasive arterial blood pressure and transcutaneous oxygen saturation monitoring. The risk of postoperative nausea and vomiting (PONV) will be assessed using a Koivuranta score, which included five risk predictors: female gender, history of PONV/motion sickness, non-smoker, anticipated postoperative opioid use and surgical time greater than 60 min [9]. Each risk predictor is scored 1 point, with a score of 0–1 as low risk, 2–3 as medium risk and 4–5 as high risk. The risk level of PONV will be recorded. Granisetron 3 mg will be used for single-drug prophylaxis in low-risk patients, and granisetron 3 mg plus dexamethasone 4 mg will be used for combination prophylaxis in middle-high-risk patients.

##### Grouping

The groupings will be numbered sequentially and sealed in opaque envelopes by an independent person not involved in this study. Once participants are in the surgical preparation room, the appropriate serially numbered envelope will be opened by a trained operating room nurse not involved in the study to identify the grouping and the nerve block will be completed according to the grouping. Another researcher who is unaware of the grouping will measure the extent of the nerve block and record relevant data.

##### Description for Intervention

Eligible participants will be randomly allocated in equal proportions between the two groups mentioned above to receive nerve block in the surgical preparation room. Each patient will receive 5 µg of sufentanil intravenously before nerve block. Participants will be placed in the lateral position, routinely disinfected and placed on the ultrasound probe using a sterile protective sleeve. All operations will be performed using a high-frequency line array probe on an M-Turbo ultrasound machine (SonoSite Inc., USA) and a short bevel puncture needle [Contiplex D continuous plexus block kit (B. Braun, Germany), 400 mm]. All patients will use 2 mL of 2% lidocaine for skin infiltration anaesthesia, explaining to the control group patients that this is nerve block puncture pain, and blinding the control group patients accordingly. Disinfect the skin and apply a towel, wrap the ultrasound probe in a sterile bag. Under ultrasound guidance, search for the erector spinae muscle, rhomboid muscle and trapezius muscle at the T4–T5 level, and insert the needle 3 cm next to the spinous process of the T5 thoracic vertebrae. Use in-plane needle insertion technology, and confirm under ultrasound that the end of the needle is located in the deep surface of the erector spinae muscle, and the puncture is successful. Based on previous literature and pre-experiments, we will select 0.5% ropivacaine 25 mL for the ESPB at the T4–T5 level and then use an indwelling catheter (depth 5 cm) and an electronic drug injection pump set at 0.2% ropivacaine 5 ml/h for continuous infusion [9]. All patients will be observed for 30 min after completion of the block, and the level of sensory block is measured and recorded every 5 min by another anaesthetist who is unaware of the group. Patients in group E who do not experience hypoalgesia during the observation period will be considered block failures and will be excluded at the time of final data unblinding. Patients in both groups will be given patient-controlled intravenous analgesia (PCIA) until 48 h after surgery. The PCIA protocol is as follows: sufentanil 100 µg + granisetron 6 mg + normal saline diluted to 100 ml, background dose 1.5 ml/h, self-controlled single dose 1.5 ml/time, locking time 30 min. If the static VAS score is ≥ 4, then parecoxib sodium 40 mg intravenous injection will be administered for rescue analgesia.

##### Introduction for investigators

The study will be done with the joint participation of the Anaesthesia Department, the Breast Surgery Department, the Central Laboratory and the Operating Theatre Nursing Department. The Anaesthesia Department participants in the study have extensive experience in nerve blocks and have received specific training in ultrasound-guided ESPB.

### Criteria for discontinuing or modifying allocated interventions {11b}

Research interventions have been identified. Once an intervention is implemented, it cannot be modified.

The investigator will discontinue the participant if one of the following occurs during the course of the experiment:

1. Participants request termination of the intervention during the course of the experiment.
2. Attempt nerve block puncture operation ≥ 3 times.
3. An unacceptable risk of a serious adverse event.
4. For various reasons, post-operative follow-up cannot be completed.

### Strategies to improve adherence to interventions {11c}

We will fix a researcher to perform the pre-surgical assessment and sign the informed consent form. The researcher will conduct a pre-anaesthetic assessment the day before the procedure, with strict exclusion and inclusion criteria. During the process of obtaining informed consent from the participants, the researcher will explain in detail what the study is about and the need for cooperation. In addition, another researcher who is unaware of the grouping will administer a brief 3–5-min questionnaire to participants within 48 h of the procedure without unduly interrupting their rest time.

### Relevant concomitant care permitted or prohibited during the trial {11d}

All study participants will receive standard post-operative care in the operating room, post-anaesthesia care unit (PACU) and wards.

### Provisions for post-trial care {30}

At the end of the trial intervention, participants will be removed from the LA injection catheter in the safe and comfortable environment of the ward and will be closely observed. Be alert to any risks associated with the study. The follow-up 8 h after the end of infusion will focus on LA-toxicity assessment. In the event of any adverse events, appropriate care and treatment will be provided by our study team and the hospital.

### Outcomes {12}

#### Primary outcome

The primary outcome is the cumulative PCA consumption, including the sum of background dose and self-control dose.

#### Secondary outcomes

1. The static and dynamic pain scores at 2, 6, 12, 24 and 48 h postoperatively. Pain is scored using a visual analogue scale (VAS), with a score of 0–10 representing pain levels ranging from no pain at all to intolerably severe pain. Dynamic pain is the pain felt when the arm is externally rotated 45° on the side of the operation.
2. The occurrence of PMPS. Defined as chronic pain not related to incision healing, which may be burning pain, pins and needles, tenderness-induced pain, or deep dull pain at the surgical or surgery-related site, and which occurs for at least 4 days in a week after surgery [10, 11].
3. The levels of inflammation-related cytokines. Concentrations of CRP, IL-6, IL-10 and TNF-α in venous blood 1 day before, 2, 6, 12, 24 and 48 h after surgery.
4. Concentration of ropivacaine in venous blood 1, 2 and 3 days after surgery.
5. Total consumption of additional analgesics. Remedial analgesia using parecoxib sodium dosage and the use of additional analgesics during the entire 12-month follow-up period.
6. Post-operative adverse reactions. Including but not limited to PONV, drowsiness, itching, respiratory depression and urinary retention.
7. Post-puncture adverse reactions. Including but not limited to haematoma, infection, nerve damage and abnormal sensation in the blocked area.
8. Postoperative recovery, including the length of stay in PACU, time of first ambulation, intake of food, voiding, gastrointestinal function, length of stay in hospital and the quality of Recovery-15 (QoR-15) questionnaire at 24 h and 48 h after surgery and on the day of discharge [9]. Discharge criteria: vital signs are normal; wound healing is good, with no obvious postoperative complications, such as wound infection, flap necrosis, or fluid accumulation due to poor drainage; pain is mild and does not require medication or oral analgesic medication to achieve satisfactory analgesia; gastrointestinal function is normal; able to get out of bed and move around freely; intravenous fluids are not required; and the patient is willing and wants to be discharged from the hospital.

### Participant timeline {13}

**Fig. 1:**
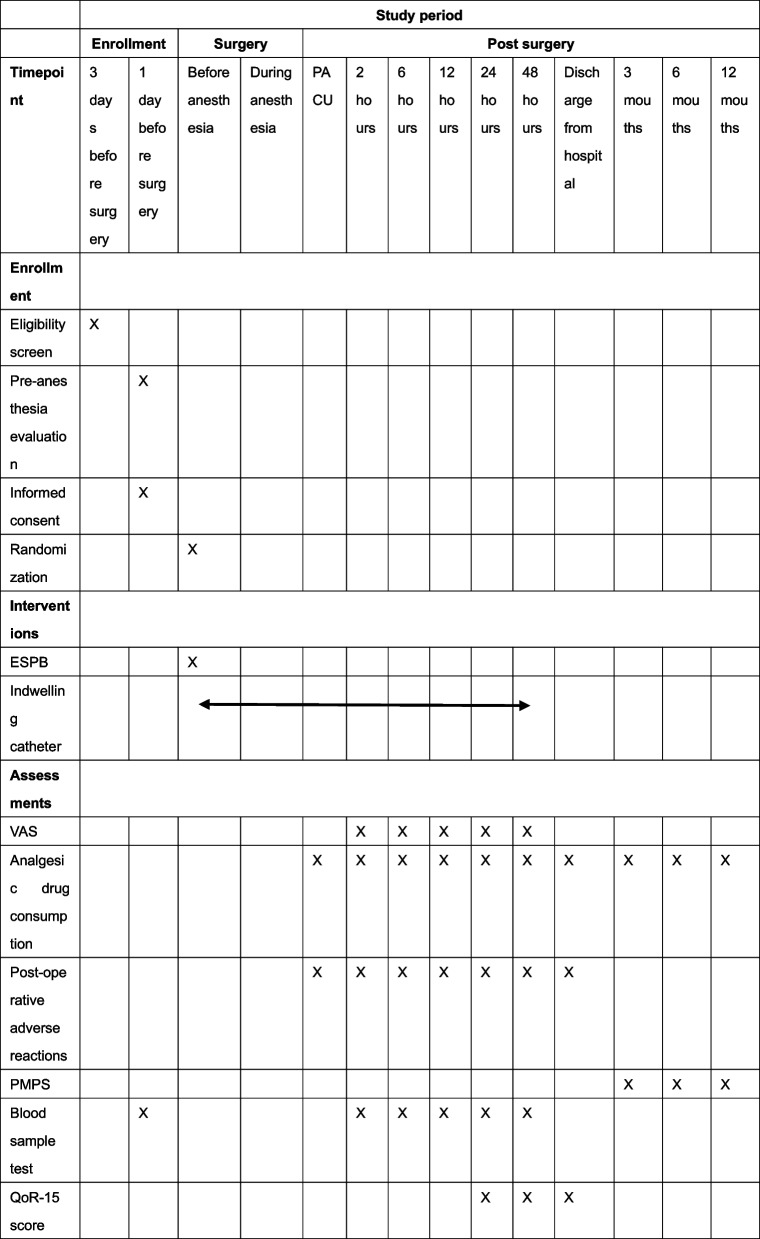
The participant timeline is shown in Fig. 1 Time schedule of enrolment, interventions, assessments and visits for participants. PACU, post-anaesthesia care unit; ESPB, erector spinae plane block; VAS, visual analogue scale; PMPS, post-mastectomy pain syndrome; QoR-15, Quality of Recovery-15 questionnaire

### Sample size {14}

The focus of this trial is to look at post-operative pain in the presence of a continuous infusion of LA. Therefore, the expected sample size is calculated based on post-surgical pain scores. Considering that ethnic and geographical differences may have some influence on the perception of pain, we chose the results of a study at another hospital that is geographically close to the location of the current study unit as the basis for the sample size calculation. Based on the results of this study and our pre-experiment, the dynamic VAS at 48 h after MRM surgery is 4.2 ± 1.4 (mean ± standard deviation) for the control group and 3.6 ± 1.1 for the ESPB group [12]. We chose *α* = 0.05, test validity *β* = 80%, and calculated 71 cases per group using PASS 15.0 software. This study is a single-centre study and there may be a degree of bias if the sample size is small, so the shedding rate needed to be increased and is set at 11%. The final sample size is extrapolated to 80 cases per group, including a total of 160 participants.

### Recruitment {15}

Our hospital provides healthcare to over 4 million people in the surrounding area. On average, more than 270 breast cancer surgeries are performed each year. Recruitment for this study begins in October 2022 and is expected to continue until 2024. A sufficient source of patients is available to ensure the completion of the recruitment of 160 eligible participants. The trial is currently in the recruitment phase and patients will be screened according to strict recruitment criteria. Prospective participants will be sourced from a surgical waiting list. Screening will be done by reviewing their health records to determine eligibility. Patients will be recruited after admission to the hospital and 1 day prior to surgery.

## Assignment of interventions: allocation

### Sequence generation {16a}

Random sequences are generated on a 1:1 basis using a computerised random number generator and researchers placed the random sequences in sealed, opaque and sequentially numbered envelopes. When participants are eligible for the study and allowed into the surgical preparation room, the researcher will open the envelope to obtain the random sequence that determined the grouping. Each participant will correspond to a unique random sequence number in an envelope in the order in which they participated in this study. The numbered information of all participants will be recorded in the randomised list.

### Concealment mechanism {16b}

The participant’s unique randomised serial number and group allocation information will be printed on a separate sheet of paper and stored confidentially. The person performing the randomisation assignment and the nerve block operator will not be involved in the subsequent study process.

### Implementation {16c}

The randomisation described above will determine whether to perform a nerve block or a sham nerve block. Once participants enter the surgical preparation room to determine the grouping, the researcher will perform the nerve block based on the grouping. The person responsible for the randomisation allocation will carry the envelope of the randomised sequence with the nurse who is not involved in the study to reveal the results of the grouping of participants.

## Assignment of interventions: blinding

### Who will be blinded {17a}

Due to the nature of the intervention in this study, the person responsible for the randomisation process and the nerve block operator will be the unblinded study personnel. Therefore, participants, anaesthetists, surgeons, post-operative follow-up staff and nerve block effect assessors will be unaware of the random allocation sequence.

### Procedure for unblinding if needed {17b}

To enable investigators to know the study group of participants in the event of an emergency, a 24-h unblinding telephone number is available. In the event of a medical emergency that may be relevant to this trial, the need for unblinding will be discussed by the principal investigator and, if indeed necessary, a rapid emergency unblinding can be carried out via the unblinding telephone number. The study team leader and ethics committee must be informed as soon as possible after the unblinding has taken place. The time, reason and outcome of the unblinding must be documented in the source document.

## Data collection and management

### Plans for assessment and collection of outcomes {18a}

Data will be collected and recorded on a case record form (CRF) at predetermined points in time. A pre-anaesthetic assessment will be conducted by the principal investigator (LY) and informed consent will be obtained from the participants 1 day prior to surgery. Two anaesthetists within the study group will perform the nerve block and assess the effect of the nerve block and record the assessment separately. The anaesthetist in the operating room is responsible for recording the medication administered during surgery. The post-operative follow-up staff and data analysts completed their respective tasks according to the study plan. The participants’ grouping is not known to anyone other than the nerve block operator. LY was the emergency contact and coordinator.

Pre-operative CRF will be completed the day before the procedure to determine eligible participants and then take venous blood and collect basic patient information and past medical history. Attention should be paid to the evaluation and prevention of PONV before surgery. The intraoperative CRF will focus on recording the dosage of anaesthetic drugs and the number, type and reason for the use of vasoactive drugs.

Post-operative CRF was completed by blinded investigators at 48 h post-operatively and on the day of discharge, focusing on recording post-operative analgesic consumption within 48 h of surgery, as well as recording VAS pain scores, adverse effects, the QoR-15 score and collection of venous blood. Follow-up and recording of the occurrence of PMPS and additional analgesics will be done at 3, 6 and 12 months after surgery.

Data analysts not involved in the clinical trial will independently analyse all data collected after the last participant has completed the trial. Compared to group C, we anticipate that continuous ESPB will reduce post-surgical pain levels, thereby reducing analgesic drug requirements and analgesic drug-related side effects, down-regulating the degree of inflammatory response and accelerating post-surgical recovery. In addition, we are not limited to pain and recovery during hospitalisation, but also focus on the incidence of chronic pain after surgery, which is more in line with the concept of enhancing the overall post-surgical recovery of patients.

### Plans to promote participant retention and complete follow-up {18b}

Participants will be fully informed and educated by the investigators in a variety of ways to understand the purpose of the trial and the benefits of accelerated recovery. Rationalise the process to minimise the time patients have to wait in the operating room. Ensure that participants have a relatively quiet environment for ESPB and follow-up visits, which will be limited to 3–5 min to protect patient privacy and rest. During the course of the trial, subjects are made aware of the possible adverse reactions to avoid shedding due to minor or normal adverse reactions. Strengthen the training of investigators who have a thorough understanding of the clinical trial, are familiar with the trial protocol, can answer patients' questions accurately and reasonably and establish a good doctor-patient relationship, thus making patients more comfortable with the trial.

Participants have the right to withdraw from the study at any time for any reason. Reasons for withdrawal will be asked, measured and recorded in the source documents, and the Ethics Committee will be informed.

### Data management {19}

This study will manage data in accordance with the Data Security Law of People’s Republic of China and the European Union’s General Data Protection Regulation [13]. Follow the principles of “clear purpose” and “minimum collection”. All research data will be filled in manually in paper CRFs. CRFs recording the data will be kept in a locked safe and then transcribed into Microsoft Excel by researchers not involved in the implementation of the intervention, who will use an offline computer to aggregate and analyse the data. To improve the quality and accuracy of the data, a data monitoring team consisting of an anaesthetist, a statistician and a nurse was set up. All data are checked for errors and those suspected of being incorrect are re-verified. Access to safe and computer is restricted to researchers assigned to the work of data entry, processing and analysis.

### Confidentiality {27}

During the trial, all paper patient information will be stored in strict confidentiality in a secure safe. All electronic information relating to the study will be stored offline on a computer. In order to protect the privacy of participants, any access to the safe and the computer will be strictly reviewed and authorised before viewing. Participants’ research information will not be used for purposes other than research without written permission. Anonymous trial data may only be shared with other researchers with the author’s consent and only for research purposes.

### Plans for collection, laboratory evaluation and storage of biological specimens for genetic or molecular analysis in this trial/future use {33}

Blood specimens collected for this study will be managed in accordance with the ethical and legal requirements of local and the International Organization for Standardization [14]. In order to determine the potential beneficial effect of continuous ESPB in reducing the inflammatory response after MRM surgery, participants will be studied for inflammation-related cytokines. Blood levels of inflammatory cytokines will be measured 1 day before surgery, 2, 6, 12, 24 and 48 h after surgery. Cytokines to be measured include pro-inflammatory cytokines (TNF-α, IL-6) and anti-inflammatory cytokines (IL-10). From a safety perspective, plasma concentrations of ropivacaine will be analysed on days 1, 2 and 3 after surgery. All blood samples will be sent to our central laboratory promptly after acquisition and centrifuged within 1 h, and plasma will be separated and stored at − 80 °C for analysis later in the trial. All plasma samples will be discarded upon completion of the study.

## Statistical methods

### Statistical methods for primary and secondary outcomes {20a}

Statistical analysis will be performed using SPSS statistical software version 27.0 (IBM). All statistical tests will be two-tailed and the significance level will be set at 0.05. Primary and secondary outcomes will be analysed using the following statistical method: the Kolmogorov–Smirnov test will be used to determine whether continuous data follow a normal distribution, and the Levene’s test was used to assess the homogeneity of variance. Normally distributed continuous data are expressed as mean ± standard deviation and compared using the independent samples* t*-test. Non-normally distributed continuous data will be expressed as median and interquartile range and compared by the Mann–Whitney *U* test. Count data is expressed as the number of cases (rate) by the chi-square test or Fisher’s exact test.

### Interim analyses {21b}

Not applicable; no interim analysis is planned for this study.

### Methods for additional analyses (e.g. subgroup analyses) {20b}

Not applicable, no additional analysis is planned for this study.

### Methods in analysis to handle protocol non-adherence and any statistical methods to handle missing data {20c}

This is an intention-to-treat study and we will explain the intervention in detail and emphasise matters of cooperation to patients during the pre-anaesthetic assessment and during obtaining informed consent, we suspect that few patients will offer non-compliance with the protocol. In the event of missing data, we will complete the data set by using multiple compensation methods as recommended by the statistical experts Hsu et al. [15] and will assess its effect by sensitivity analysis. We anticipate that few patients will be lost to follow-up due to the nature of clinical practice of the current trial intervention.

### Plans to give access to the full protocol, participant-level data and statistical code {31c}

This is a principal investigator-initiated trial. Access to the full protocol, participant-level data and statistical code for research purposes will be considered upon submission of a reasonable written request and obtaining authorization from the principal investigator.

## Oversight and monitoring

### Composition of the coordinating centre and trial steering committee {5d}

This is a single centre trial and there will be no coordinating centre or trial steering committee. The principal investigator will hold weekly study team meetings to discuss and analyse the progress of the study and to report any serious incidents to the Ethics Committee. There will be no stakeholder or private sector involvement.

### Composition of the data monitoring committee, its role and reporting structure {21a}

We expect to rapidly complete the clinical intervention trial, there is no evidence of significant safety concerns with this study intervention, the participants are not involved in specific disease groups or life-threatening conditions and therefore no specific data monitoring committee will be established. The “data monitoring team” involved in item 19 is responsible for verifying source documents and possible adverse events, and the membership of this team and the process of verification will be independent of the study process.

### Adverse event reporting and harms {22}

Adverse events, adverse drug reactions, unexpected adverse drug reactions, and serious adverse drug reactions will be defined according to the guidelines for good clinical practice of the Quality Management Standards for Drug Clinical Trials in China [16]. We will assess the nature (expected versus unintended), severity (serious versus non-serious) and relevance to the intervention (relevant versus irrelevant) of each adverse event. Serious, unintended and intervention-related adverse events will be reported to the Ethics Committee. The principal investigator will conduct regular reviews of all adverse events and convene adverse event assessment discussion meetings as necessary. Any adverse events that occur in this trial will be recorded on the CRF and reported to the principal investigator. In the meantime, subjects will be followed until they are deemed to have fully recovered or overcome the adverse event.

## Frequency and plans for auditing trial conduct {23}

A nurse who is not involved in this trial will act as an independent reviewer for the duration of the trial. Independent review will be conducted every 2 weeks or after every 10 newly recruited participants have completed a 48-h post-operative follow-up. All errors will be recorded and reported to the principal investigator. The audit will include a review of CRF content, missing data, duplicate data, incorrect data and informed consent documentation.

### Plans for communicating important protocol amendments to relevant parties (e.g. trial participants, ethical committees) {25}

The Ethics Committee has reviewed the protocol of this study and has given its consent for the study protocol to be carried out. No amendments can be made to the study protocol unless permission is obtained from the Ethics Committee. If a protocol amendment is indeed necessary, it should be reviewed by the principal investigator and a written request for the amendment should be made, and the written request for the amendment will be submitted to the Ethics Committee for further review and approval.

## Dissemination plans {31a}

In accordance with standard protocol guidelines, the authors state that unblinding data from the trial will not be available until the primary outcomes are published. Deblinding will take place at the end of the study. A clinical article will be written on the primary and secondary outcomes of the study and every effort will be made to publish the results in peer-reviewed journals related to clinical anaesthesia and breast surgery, and the results will be disseminated regardless of the size or direction of impact.

## Discussion

Post-operative pain is the most common and urgent acute pain in clinical practice, including incisional pain and inflammatory pain due to surgical trauma, but the control of acute post-operative pain is still unsatisfactory [17]. If acute postoperative pain is not adequately controlled in its initial state, it may develop into chronic postoperative pain and subsequently lead to an inflammatory response, seriously affecting the patient’s postoperative quality of life and increasing the risk of postoperative complications, which in turn affects the patient’s rapid postoperative recovery. Perfect perioperative analgesia is therefore essential.

Regional nerve block techniques, an important component of multimodal analgesia, were used in various types of post-surgical analgesia with definite results. Regional nerve block techniques commonly used in clinical practice for breast surgery include epidural anaesthesia and thoracic paravertebral nerve block, but epidural anaesthesia carries the risk of spinal cord injury and epidural haematoma and is contraindicated in patients with coagulation abnormalities or on anticoagulant medication, and thoracic paravertebral block carries the risk of pneumothorax [18]. Both of these analgesic modalities are difficult to perform and have a high failure rate, making them difficult for beginners to master. Therefore, the search for an effective, simple and safe regional nerve block technique for post-breast surgery analgesia becomes vital. The most significant advantage of the ESPB is that it is simple and safe to perform, and the puncture route is free of vital vessels and organs. The images of the T5 transverse process and the muscle gap are easily identified during the ultrasound-guided procedure [19, 20]. Thus, the ESPB is more feasible than the above two blocking techniques.

Although ESPB was used with definite effectiveness for post-surgical analgesia, most clinical applications were currently for a single injection of LA [7–9]. Theoretically, the maximum duration of a single dose of LA is 12 h [21]. In contrast, continuous administration of the LA after placement of the catheter can provide a longer duration of analgesia. A multicentre, high-quality RCT showed that nerve block with a single injection of LA improved acute pain after breast cancer surgery but did not reduce the incidence of chronic pain [22]. There are still few studies on the use of continuous ESPB in analgesia for MRM surgery, and our research on this occasion is much needed.

Poorly controlled acute pain after surgery is an important contributor to chronic pain and stress and inflammatory responses [23]. We believe that improving acute pain within 48 h of surgery is even more important and therefore used it as the primary outcome for this study. We also evaluated the postoperative recovery in a multidimensional manner by comparing the incidence of PMPS, the QoR-15 score, the concentration of perioperative inflammatory cytokines, total consumption of analgesic agents and post-surgical adverse effects.

In summary, our single-centre, prospective, double-blind RCT will reveal the effect of continuous ESPB on postoperative pain and inflammatory response in patients undergoing MRM surgery and is expected to provide a strong scientific basis for its use in the management of MRM surgery postoperatively. Continuous ESPB and up to 12 months of follow-up are two innovations of this trial. In addition, our results may be extrapolated to other chest procedures and comparisons with the application of different concentrations of local anaesthetics.

## Trial status

The trial is registered on the Chinese Clinical Trial Registry (http://www.chictr.org.cn) identifier: ChiCTR2200061935. The current protocol is version 1.2 of 28 March 2023. Recruitment for the trial will begin in October 2022 and we are currently recruiting patients. Recruitment will be completed in approximately December 2023.

### Supplementary Information

**Additional file 1. **Preoperative, intraoperative and postoperative CRFs.

**Additional file 2. **Informed consent form.

**Additional file 3. **QoR-15 questionnaire.

## Data Availability

All data for this study can be obtained from the corresponding author upon reasonable request and for research purposes only.

## Abbreviations

LA: Local anaesthetic
ESPB: Erector spinae plane block
MRM: Modified radical mastectomy
Group E: ESPB group
Group C: Sham block group
PMPS: Post-mastectomy pain syndrome
ELISA: Enzyme-linked-immunosorbent serologic assay
VAS: Visual analogue scale
CRF: Case record form
PACU: Post-anaesthesia care unit
PCIA: Patient-controlled intravenous analgesia
QoR-15: Quality of Recovery-15
