# Supplementary material for: Effect of ultrasound-guided continuous erector spinae plane block on postoperative pain and inflammatory response in patients undergoing modified radical mastectomy for breast cancer: study protocol for a randomised controlled trial
Source: Trials. 2024 Jan 15;25:51. doi: 10.1186/s13063-023-07777-0 (PMC10788999; doi:10.1186/s13063-023-07777-0)
Supplement: Supplementary file 1 — Additional file 1. Preoperative, intraoperative and postoperative CRFs. [file 13063_2023_7777_MOESM1_ESM.pdf]

Preoperative CRF Name:__________ Inpatient number:__________

Age		ASA	I	
Inpatient number			II	
Surgical site(Left or Right)			III	
Contace information 1		Recent situation	NSAIDs	
Contace information 2			Steroid	
Home address			Opioids	
Weight/height(cm/kg)			Upper respiratory infections	
Preoperative diagnosis		Patient special	Hypertension	
Clinical stages			Diabetes	
History of drug allergy			Cardiovascular disease	
Any other disease history that needs to be recorded			Insomnia	
			Surgical history	

CRF: case record form; ASA score: American Society of Anesthesiologists Score; cm: centimeter; kg: kilogram; NSAIDs: non-steroidal anti-inflammatory drugs.

Intraoperative CRF   Name:__________   Inpatient number:__________

Anesthesia duration (min)		
Bleeding (ml)		
Urine volum (ml)		
Infusion type and total (ml)		
Duration of ESPB and reason for failure (min)*	Total	
	1st use time and reason	
	2nd use time and reason	
	3rd use time and reason	
Heart rate and blood pressure immediately after ESPB		
Immediate complication of ESPB		
Changes in heart rate and blood pressure and treatment measures		
Total consumption of anaesthetic drugs intraoperatively	Opioids	
	Sedative drugs	
	muscle relaxant	

CRF: case record form; min: minute; ml: milliliter; 1st: first; 2nd: second; 3rd: third; ESPB: erector spinae plane block; *Defined as the time from the ultrasound probe contacting the skin to the completion of the catheter placement.

Postoperative CRF Name:__________ Inpatient number:__________

Extubation time (min)			
PACU retention time (min)			
VAS	Time after surgery (hours)	Static pain score	Dynamic pain score※
	2		
	6		
	12		
	24		
	48		
Post-operative PMPS occurrence (mouths) ★			
3			
6			
12			
Number of PCIA presses within 48 hours of surgery			
Rescue analgesia (time, type, dosage)			
Post-operative adverse reactions			
Post-puncture adverse reactions			
Postoperative recovery	Time of first ambulation		
	Hospitalization days		
Other conditions that need to be recorded			

PACU: post-anesthesia care unit; VAS: visual analogue scale; PMPS: post-mastectomy pain syndrome; PCIA: patient controlled intravenous analgesia; ※Defined as the pain felt when the arm is externally rotated 45 degrees on the side of the operation; ★Defined as chronic pain not related to incision healing, which may be burning pain, pins and needles, tenderness induced pain or deep dull pain at the surgical or surgery related site, and which occurs for at least 4 days in a week after surgery.
