# Supplementary material for: Effect of ultrasound-guided continuous erector spinae plane block on postoperative pain and inflammatory response in patients undergoing modified radical mastectomy for breast cancer: study protocol for a randomised controlled trial
Source: Trials. 2024 Jan 15;25:51. doi: 10.1186/s13063-023-07777-0 (PMC10788999; doi:10.1186/s13063-023-07777-0)
Supplement: Supplementary file 2 — Additional file 2. Informed consent form. [file 13063_2023_7777_MOESM2_ESM.pdf]

Informed consent

Version No: 3.0 Version Date: December 10, 2021

Dear patient:

We invite you to take part in a clinical study entitled "Effect of ultrasound-guided continuous erector spinae plane block on postoperative pain and inflammatory response in patients undergoing modified radical mastectomy for breast cancer: study protocol for a randomized controlled trial". Before you decide whether to participate in this study, please read the following carefully. It can help you understand the purpose, the procedure and duration of the study, and the possible benefits, risks and inconveniences after participating in the study. If you have any questions or don't understand, please consult the researcher (doctor) until you fully understand. You can also discuss with your family, relatives, friends, etc. to help you make a decision that is in your interest.

The following is an introduction to this study:

1. Study background and purpose

Erector spinae plane block is a novel interfascial plane block technique, which has the advantages of simple operation, easy localization and high safety. Continuous erector spinae plane block had longer analgesic effect and better analgesic effect under the condition of indwelling catheter. Recent studies have shown that in many types of surgery, erector spinae plane block can effectively reduce postoperative pain and promote rapid postoperative rehabilitation. Studies have also shown the effectiveness of this technique in postoperative analgesia after breast surgery.

Principal in charge:

Yu Liang, Deputy Chief Physician of the Anesthesiology Department of Huzhou Central Hospital, Master's Candidate of the School of Medicine of Zhejiang University, and Master's Candidate Supervisor of Zhejiang Chinese Medical University.

2. Specific procedures and processes

The study is expected to have 160 participants. We will perform ultrasound-guided continuous erector spinae plane block on participants according to the study design and collect relevant clinical data:(1) Static and dynamic VAS scores at 2h,6h,12h,24h and 48h after operation;(2) Total opioid consumption of analgesia pump;(3) the rescue analgesia rate of parecoxib sodium;(4) PACU stay time;(5) Time of first ambulation;(6) Length of hospital stay;(7) Incidence of postoperative adverse reactions (such as nausea, vomiting, lethargy, pruritus, respiratory depression, urinary retention, etc.);(8) Incidence of puncture related complications (such as hematoma, infection, nerve injury, etc.);(9) Incidence of postoperative PMPS;(10) Inflammatory factors: CRP, IL-6, IL-10 and TNF-α concentrations in venous blood 1 day before operation and 2h, 6h, 12h, 24h and 48h after operation. Finally, the data of each group were analyzed statistically. A total of 1 year will be required from study participation to the end of the last follow-up visit, and telephone follow-up will be the main follow-up after discharge.

3. What do you need to do if you take part in the study

If you agree to take part in our study, please sign the informed consent form the day before the procedure. Your vital signs will be monitored routinely and you will be given an ultrasound-guided continuous erector spinae plane block prior to anesthesia. This will help reduce your post-operative pain. Please cooperate with the procedure. If in doubt, we will

give you a detailed explanation.

4. The possible benefits of participating in this study

Continuous erector spinae plane block may reduce postoperative pain after breast surgery, reduce postoperative opioid and other analgesics, reduce postoperative adverse reactions and postoperative complications, reduce postoperative inflammatory response, and thus promote rapid postoperative recovery, but we cannot guarantee this. Although taking part in this trial may not directly benefit you, your participation may benefit future patients who suffer the same pain.

You will not be paid for taking part in this study, and if you are harmed as a result of taking part in this study:In case of any damage related to the clinical trial, you can get free treatment and/or corresponding compensation, or provide compensation through relevant legal channels. If you are complicated with the treatment and examination required by other diseases, or the complications that may occur in surgery and anesthesia, that is, no matter whether you participate in this study or not, the complications that may occur as long as you receive this surgery (these complications, your surgery or the doctor in charge, anesthesiologist will tell you or your authorized agent) will not be included in the free scope.

5. Possible adverse reactions, risks, prevention and treatment measures of participating in this study

Difficulty in puncture during operation, repeated puncture;Puncture site infection; hematoma formation, nerve injury, pneumothorax, toxic reaction of local anesthetics, etc.

If you have any discomfort, new changes in your condition, or any unexpected situation

during the study, you should inform your doctor in time, and the doctor will make judgment and medical treatment. In case of the above adverse reactions related to this study, or other adverse reactions clearly caused by this study, you can get free treatment and/or corresponding compensation, or provide compensation through relevant legal channels.

6. Description of expenses

Some drugs and materials required for the study (including the narcotic drug Naropin, nerve block puncture kit and venous blood collection supplies) are free of charge. The treatment expenses arising from study-induced injuries such as complications during the study shall be borne by the research group. If you also need treatment and examination for other diseases, it will not be free of charge.

7. Alternative scheme

This study was a prospective, randomized controlled study. If you do not agree to participate in this study, you can use the current general anesthesia and postoperative analgesia measures in clinical practice.

8. Your rights

You can choose not to participate in this study, or withdraw at any time after notifying the investigator without discrimination or retaliation, and any of your medical treatment and rights and interests will not be affected. The study staff may terminate your participation in the study at any time if you need other treatment, if you do not follow the study plan, if you have a study-related injury, or for any other reason. You can keep abreast of the information materials and study progress related to this study at any time. If any new safety information related to this study is found, we will also inform you in a timely

manner. If you have any questions about this trial, or if you have any discomfort or injury during the study, or if you have any questions about the rights and interests of participants in this study, you can contact the study staff by telephone.

Your participation in this study is entirely voluntary. You can refuse to participate in this study, or withdraw from the study at any time during the study without reason, which will not affect your relationship with the doctor, nor will it affect the loss of your medical treatment or other benefits. If you have any questions about this study or have any questions during the study, please contact the investigator of this project:Yu Liang, Contact Information:13868298615.

9. Confidentiality of your personal information

Your medical records (including study medical records and physical and chemical test reports, etc.) will be kept in the hospital as required. Except for the investigator, Ethics Committee, monitoring, audit, drug administration and other relevant personnel who will be allowed to access your medical records, other personnel unrelated to the study have no right to access your medical records without permission. Public reporting of the results of this study will not disclose your personal information. We will make every effort to protect the privacy of your personal medical data to the extent permitted.

10. Termination of participation in the study

Your participation in this study may be terminated for the following reasons:

① You do not follow the study doctor's orders.

② You have a serious condition that may require treatment.

③ You withdraw your consent.

11. Ethics Committee

This study has been reported to the Medical Ethics Committee of Huzhou Central Hospital and approved by the Committee. During the study process, you can contact the Medical Ethics Committee of Huzhou Central Hospital for matters related to ethics and rights.

Tel: 0572-2709719; Email address:hzszxyyll@163.com

Researcher statement:

I confirmed that I had explained the details of this study to the patient, including his rights, possible benefits and risks, and answered his (and/or guardian/witness) questions. He (and/or guardian/witness) said that he understood my instructions and explanations. I have given him (and/or guardian/witness) a copy of the signed informed consent form.

Signature of researcher: _ _ _ _ _ _ _ _ _ _ _ _ _ _ _ _

Contact number of researcher: _ _ _ _ _ _ _ _ _ _ _ _ _ _ _ _

Date of conversation: _ _ _ _ _ _ _ _ _ _ _ _ _ _ _ _

The subject (or guardian/witness) declares that:

I confirm that I have read the informed consent form of this study. The researcher explained the relevant contents to me in detail, answered my related questions, and made me understand the terms and conditions. I also confirmed that if I did not participate in this study or quit at any time in the middle of the study, it would not affect my diagnosis and treatment activities and doctorpatient relationship, or cause other interests damage. My

personal information will not be disclosed in the public reports that I know the results of this study. I have plenty of time to think about it. After careful consideration, I decided to accept the treatment (study) methods in this study, and agreed to use my relevant study data and information for the public reports related to the results of this study.

Subject's signature: _ _ _ _ _ _ _ _ _ _ _ _ _ _ _ _ Signature date: _ _ _ _ _ _ _ _ _ _ _ _ _ _ _

Contact number of the subject: _ _ _ _ _ _ _ _ _ _ _ _ _ _ _ _

If the subject has insufficient self-knowledge or incapability, his guardian should sign it.

Guardian's signature: _ _ _ _ _ _ _ _ _ _ _ _ _ _ _ _ Signature date: _ _ _ _ _ _ _ _ _ _ _ _ _ _

Contact number of guardian: _ _ _ _ _ _ _ _ _ _ _ _ _ _ _ _

If the subject or his guardian can't read or write, the witness's signature is required.

Signature of witness: _ _ _ _ _ _ _ _ _ _ _ _ _ _ _ _ Signature date: _ _ _ _ _ _ _ _ _ _ _ _ _ _

Witness contact number: _ _ _ _ _ _ _ _ _ _ _ _ _ _ _ _
