# Supplementary material for: Effect of ultrasound-guided continuous erector spinae plane block on postoperative pain and inflammatory response in patients undergoing modified radical mastectomy for breast cancer: study protocol for a randomised controlled trial
Source: Trials. 2024 Jan 15;25:51. doi: 10.1186/s13063-023-07777-0 (PMC10788999; doi:10.1186/s13063-023-07777-0)
Supplement: Supplementary file 3 — Additional file 3. QoR-15 questionnaire. [file 13063_2023_7777_MOESM3_ESM.pdf]

ChiCTR2200061935 版本V1.2 版本创建时间2023/3/28 21:33:16 中国临床试验注册中心

审核状态： **通过审核**
Project audit state: Successful

注册号：<br>Registration number:	ChiCTR2200061935
最近更新时间：<br>Date of Last Refreshed on:	2023/3/28 10:30:35
注册号状态：	预注册
Registration Status:	Prospective registration
填写语言：	中文和英文
Language:	Chinese And English
注册题目：	超声引导下连续竖脊肌平面阻滞对乳腺癌改良根治术患者术后疼痛及炎症反应的影响
Public title:	Effect of ultrasound-guided continuous erector spinae plane block on postoperative pain and inflammatory response in patients undergoing modified radical mastectomy for breast cancer
注册题目简写：	
Public title acronym:	
研究课题的正式科学名称：	超声引导下连续竖脊肌平面阻滞对乳腺癌改良根治术患者术后疼痛及炎症反应的影响
Scientific title:	Effect of ultrasound-guided continuous erector spinae plane block on postoperative pain and inflammatory response in patients undergoing modified radical mastectomy for breast cancer
研究课题的正式科学名称简写：	
Scientific title acronym:	
研究课题代号(代码)：<br>Study subject ID:	
在其它机构的注册号：<br>Secondary ID:	

申请注册联系人：	余亮	研究负责人：	余亮
Applicant:	Yu Liang	Study leader:	Yu Liang
申请注册联系人电话：<br>Applicant telephone:	13868298615	研究负责人电话：<br>Study leader's telephone:	13868298615
申请注册联系人传真 ：<br>Applicant Fax:		研究负责人传真：<br>Study leader's fax:	
申请注册联系人电子邮件：<br>Applicant E-mail:	cangxingyulian@163.com	研究负责人电子邮件：<br>Study leader's E-mail:	cangxingyulian@163.com
申请单位网址(自愿提供)：<br>Applicant website(voluntary supply):		研究负责人网址(自愿提供)：<br>Study leader's website(voluntary supply):	
申请注册联系人通讯地址：	浙江省湖州市吴兴区三环北路1558号湖州市中心医院6号楼5楼麻醉科	研究负责人通讯地址：	浙江省湖州市吴兴区三环北路1558号湖州市中心医院6号楼5楼麻醉科
Applicant address:	Department of Anesthesiology, 5th floor,Building 6, Huzhou Central Hospital, 1558 Sanhuan North Road, Wuxing District, Huzhou City, Zhejiang Province	Study leader's address:	Department of Anesthesiology, 5th floor,Building 6, Huzhou Central Hospital, 1558 Sanhuan North Road, Wuxing District, Huzhou City, Zhejiang Province
申请注册联系人邮政编码：<br>Applicant postcode:		研究负责人邮政编码：<br>Study leader's postcode:	
申请人所在单位：	湖州市中心医院		
Applicant's institution:	Huzhou Central Hospital		

是否获伦理委员会批准：	是		
Approved by ethic committee:	Yes		
伦理委员会批件文号：<br>Approved No. of ethic committee:	202112016-01	伦理委员会批件附件：<br>Approved file of Ethical Committee:	查看附件View
批准本研究的伦理委员会名称：	湖州市中心医院医学伦理委员会		
Name of the ethic committee:	Medical Ethics Committee of Huzhou Central Hospital		
伦理委员会批准日期：<br>Date of approved by ethic committee:	2021-12-16		
国家FDA批准文号：<br>Approved No. of SFDA:		国家FDA批准附件：<br>Approved file of SFDA:	
国家FDA批准日期：			

Date of approved by SFDA:

研究实施负责（组长）单位:	湖州市中心医院
Primary sponsor:	Huzhou Central Hospital
研究实施负责（组长）单位地址:	湖州市中心医院
Primary sponsor's address:	Huzhou Central Hospital

试验主办单位(项目批准或申办者): Secondary sponsor:

国家:	中国	省(直辖市):	浙江	市(区县):	湖州
Country:	China	Province:	Zhejiang	City:	Huzhou
单位(医院):	湖州市中心医院	具体地址:	吴兴区三环北路1558号		
Institution hospital:	Huzhou Central Hospital	Address:	1558 Third Ring North Road, Wuxing District		

经费或物资来源:	湖州市科学技术局立项项目
Source(s) of funding:	Project approved by Huzhou science and Technology Bureau
研究疾病:	乳腺癌改良根治术患者术后疼痛及炎症反应
Target disease:	postoperative pain and inflammatory response in patients undergoing modified radical mastectomy for breast cancer
研究疾病代码:	
Target disease code:	
研究类型:	干预性研究
Study type:	Interventional study
研究所处阶段:	治疗新技术临床试验
Study phase:	New Treatment Measure Clinical Study
研究目的:	证实超声引导下连续竖脊肌平面阻滞应用于乳腺癌改良根治术患者术后镇痛的效果，分析其对乳腺癌术后疼痛综合征发生率的影响，并通过检测外周静脉血中炎性因子的浓度，研究其对乳腺癌术后炎症反应的影响。以期为乳腺癌手术患者提供更为简单、安全、有效的围术期镇痛方法，为竖脊肌平面阻滞在临床中的进一步推广应用提供新的证据。
Objectives of Study:	To confirm the effect of ultrasound-guided continuous erector spinae plane block on postoperative analgesia in patients undergoing modified radical mastectomy for breast cancer, analyze its impact on the incidence of post-mastectomy pain syndrome, and study its impact on postoperative inflammatory response in breast cancer by detecting the concentration of inflammatory factors in peripheral venous blood. In order to provide more simple, safe and effective perioperative analgesia for patients with breast cancer, and provide new evidence for the further promotion and application of erector spinalis block in clinical practice.
研究设计:	随机平行对照
Study design:	Parallel
纳入标准:	年龄18-80岁，ASA I-Ⅱ级，无心、肝、肾和内分泌疾病，肺功能正常或轻度减退，术前均未接受放疗、化疗以及辅助免疫治疗的患者。手术均采用一侧乳腺根治性切除合并腋窝淋巴结清扫。
Inclusion criteria	Patients aged 18-80 years, ASA I-II, without heart, liver, kidney and endocrine diseases, normal or slightly decreased lung function, and without preoperative radiotherapy, chemotherapy and adjuvant immunotherapy. Radical mastectomy with axillary lymph node dissection was performed in all cases.
排除标准:	①竖脊肌平面阻滞禁忌患者；②乳腺二次手术患者；③严重脏器功能衰竭的患者；④参加其他临床干预试验的患者；⑤有精神病史的患者；⑥病态肥胖（BMI>40kg/m2）的患者；⑦有麻醉药物滥用史的患者。
Exclusion criteria:	① Contraindication of erector spinalis plane block; ② Patients with secondary breast surgery; ③ Patients with severe organ failure; ④ Patients participating in other clinical intervention trials; ⑤ Patients with mental history; ⑥ Morbidly obese patients (bmi>40kg/m2); ⑦ Patients with a history of narcotic drug abuse.
研究实施时间: Study execute time:	从From2022-10-10至To 2024-09-30

干预措施: Interventions:

组别:	试验组	样本量:	80
Group:	Experimental group	Sample size:	
干预措施:	连续竖脊肌平面阻滞联合全身麻醉	干预措施代码:	
Intervention:	Continuous erector spinae plane block combined with general anaesthesia	Intervention code:	
组别:	对照组	样本量:	80
Group:	Control group	Sample size:	
干预措施:	假阻滞联合全身麻醉	干预措施代码:	
Intervention:	Sham block combined with general anaesthesia	Intervention code:	

研究实施地点: Countries of recruitment and research settings:

国家:	中国	省(直辖市):	浙江	市(区县):	湖州
Country:	China	Province:	Zhejiang	City:	Huzhou
单位(医院):	湖州市中心医院	单位级别:	三级甲等		
Institution	Huzhou Central Hospital	Level of the	Tertiary A		

hospital： institution：

测量指标：
Outcomes：

指标中文名：	疼痛视觉模拟评分	指标类型：	主要指标
Outcome：	Visual Analogue Scale (VAS) score	Type：	Primary indicator
测量时间点：		测量方法：	
Measure time point of outcome：		Measure method：	
指标中文名：	镇痛药物需求量	指标类型：	次要指标
Outcome：	Analgesic drug demand	Type：	Secondary indicator
测量时间点：		测量方法：	
Measure time point of outcome：		Measure method：	
指标中文名：	术后不良反应	指标类型：	次要指标
Outcome：	Postoperative adverse reactions	Type：	Secondary indicator
测量时间点：		测量方法：	
Measure time point of outcome：		Measure method：	
指标中文名：	乳腺癌术后疼痛综合征发生率	指标类型：	次要指标
Outcome：	Incidence of post-mastectomy pain syndrome	Type：	Secondary indicator
测量时间点：		测量方法：	
Measure time point of outcome：		Measure method：	
指标中文名：	炎症因子	指标类型：	次要指标
Outcome：	Inflammatory cytokines	Type：	Secondary indicator
测量时间点：		测量方法：	
Measure time point of outcome：		Measure method：	

采集人体标本：
Collecting sample(s) from participants：

标本中文名：	血液	组织：	
Sample Name：	Blood	Tissue：	
人体标本去向	使用后销毁	说明	
Fate of sample：	Destruction after use	Note：	

征募研究对象情况： 尚未开始
Recruiting status： Pending

年龄范围：
Participant age：

最小 Min age	18	岁 years
最大 Max age	80	岁 years

性别： 女性

Gender： Female

随机方法（请说明由何人用什么方法产生随机序列）： 使用计算机化的随机数生成器以1：1的基础生成随机序列，调查人员将随机序列放入密封、不透明和按顺序编号的信封中。当参与者被允许进入手术室时，调查员将打开信封以获得确定分组的随机序列。每个参与者都将按照他们参与这项研究的顺序在信封上得到一个唯一的研究序列号。所有参与者的随机序列及其对应的研究序列号将记录在随机化列表中。

Randomization Procedure (please state who generates the random number sequence and by what method)： A computerised random number generator is used to generate a random sequence on a 1:1 basis, which is placed by the investigator in sealed, opaque and sequentially numbered envelopes. Each participant will be given a unique study sequence number.

UTN(全球唯一识别码)：

盲法： 参与者、手术间内的麻醉医生、外科医生、术后随访人员和神经阻滞效果评估员将被设盲，不知道随机分配序列。由于本研究中干预的性质，负责随机化过程的人员和神经阻滞操作员将是非盲研究人员，他们将不参加随后的研究过程。

Blinding： Participants, anesthesiologists in the operating room, surgeons, post-operative follow-up staff and nerve block effect assessors will be blinded to the sequence of random assignment. Due to the nature of the intervention in this study, the person responsible for the randomisation process and the nerve block operator will be non-blinded study personnel who will not participate in the subsequent study process.

试验完成后的统计结果：

Calculated Results ater the Study Completed：

研究负责(组长)单位：

Organizer institution (leader institution)：

资料收集汇总单位： 0

Data collection Institution：

资料管理单位：	这是一项由主要研究者发起的试验。在提交合理的书面申请并获得主要研究者的授权后，将考虑为研究目的分享原始数据。
Data management Institution：	This is a principal investigator-initiated trial. The sharing of raw data for research purposes will be considered upon submission of a reasonable written request and authorization from the principal
资料分析单位：	使用标准化的数据收集和管理系统包括纸质的病例记录表，使用ResMan平台，对试验数据进行过程质控。
Data analysis Institution：	The standardized data collection and management system, including the paper case record form, and ResMan platform are used to perform process quality control on the test data.
